# Lower metacognitive abilities predict procrastination: a cross-sectional study of a healthy adult sample

**DOI:** 10.1186/s40359-026-04221-1

**Published:** 2026-02-25

**Authors:** Rannveig Grøm Sæle, Per Matti Aslaksen, Frode Svartdal, Marte Christine Ørbo

**Affiliations:** 1 Department of Psychology, The Faculty of Health Sciences, UiT The Arctic University of Norway, Tromsø, Norway; 2 Department of Child and Adolescent Psychiatry, Division of Child and Adolescent Health, University Hospital of North Norway, Tromsø, Norway

**Keywords:** Procrastination, Executive functions, Self-regulation, Performance-Based Tests, Self-Report.

## Abstract

**Background:**

Procrastination, the tendency to delay tasks despite negative consequences, is a widespread issue linked to poor mental health and reduced daily functioning. While often attributed to self-regulation failures, the specific cognitive mechanisms underlying procrastination remain unclear. This study investigates the role of executive functions (EF), measured through both self-report and performance-based tests, in predicting procrastination in adults.

**Methods:**

A cross-sectional study was conducted with 108 adults (22–65 years) using the Irrational Procrastination Scale (IPS) to measure procrastination. EF was assessed using the self-report measure Behavior Rating Inventory of Executive Function-Adult version (BRIEF-A), which includes nine subscales organized in three factors (Behavior Regulation, Emotional Regulation, and Metacognition), and five performance-based tests assessing inhibition, working memory, cognitive flexibility, verbal fluency, and problem solving. Conscientiousness, neuroticism, depression, and rumination were included as covariates in a hierarchical multiple regression analysis.

**Results:**

Performance-based EF tests showed that participants performed within the normal to high average range across measures, indicating no clinically significant executive impairments. The BRIEF-A T-scores also reflected average executive functioning across all subscales. However, slightly elevated scores were observed on the Initiate and Working Memory subscales. The BRIEF-A metacognitive factor predicted procrastination best, in particular the Initiate subscale. Conscientiousness and age were also significant predictors, and the final model explained 55% of the variance. Self-reported problems on indices for Emotional and Behavior regulation correlated significantly positively with procrastination, but rendered non-significant when included together with the meta-cognitive index in a regression model. Performance-based EF tests showed no significant associations with procrastination.

**Conclusions:**

The findings highlight the importance of metacognitive abilities in understanding procrastination, particularly problems with task initiation. The lack of association with performance-based EF tests underscores the need for real-world measures of EF in procrastination research.

## Background

Procrastination, the tendency to delay planned tasks despite expected negative consequences, is a pervasive phenomenon across age groups and contexts [1], and is linked to poorer mental health, lower quality of life, increased stress, and reduced daily functioning [1–4]. It is primarily viewed as a failure of self-regulation [5], and in particular, there is a gap between the intention to act and the action itself [6]. Yet the specific cognitive processes involved remain insufficiently understood and have received limited empirical attention. The present study addresses this gap by investigating executive functions as predictors of procrastination in a diverse adult sample.

Two of the most influential models in modern procrastination research are the Temporal Motivation Theory (TMT) [7, 8] and the Mood Repair Model [9, 3]. Both models emphasize poor self-regulation and deficient cognitive control as key mechanisms [5]. TMT is based on an expectancy-value framework and posits that procrastination occurs when task rewards are distant in time, and the individual exhibits low expectancy of success. As a result, motivation to pursue long-term goals is reduced, paving the way for immediate temptations of lower value, especially when the task is perceived as aversive or demanding [10]. The Mood Repair Model focuses on short-term emotional relief, where individuals procrastinate to avoid or escape the negative emotions associated with the task, such as anxiety or boredom. This leads to a short-term mood improvement but reinforces the problem in the long run [3]. Both models imply that cognitive control is reduced when outcomes are delayed. According to the TMT model this occurs because value is lower when being distant in time, i.e., temporal discounting [7], and because task aversiveness directly reduces it (e.g., [1])- The mood repair model attributes this reduction to procrastinators being present-oriented [11].

These models fit within a dual-process theoretical framework of self-regulation, where human behavior is governed by two systems: a fast, impulsive, and emotion-driven system (“System 1”, or “hot” processes), and a slower, reflective, and controlled system (“System 2”, or “cold” processes) (e.g., [12–16]). The conflict between hot and cold processes is at the core of dual-process models [17], and the impact of impulsiveness relative to reflectiveness increases with higher cognitive load and poorer working memory capacity [12, 16]. In the Process Model of Self-Control [18], proactive strategy use is emphasized to prevent or avoid these conflicts [18, 17, 19]. Such strategies can include manipulating *cognition*, such as directing attention away from the temptation, or thinking about positive aspects of restraining it or negative consequences of giving in. However, they may also involve choosing or manipulating the *situation* to avoid the temptation to appear in the first place (e.g., putting the phone far away to avoid scrolling when the goal is to read a book or clean the apartment).

Executive functions (EF) refer to a set of higher-order cognitive processes involved in planning, monitoring, impulse control, cognitive flexibility, and goal-directed behavior [20–23]. EFs are multifaceted and complex to measure, which presents methodological challenges [23, 24]. In clinical psychological practice, standardized performance-based neurocognitive tests are considered the gold standard for measuring EF under standardized conditions, as they provide information on what constitutes normal performance relative to age, are well-documented in terms of psychometric properties, and have a large body of empirical evidence regarding their underlying brain correlates. In comparison, self-reported measures of daily life problems with behavior representative of EF capture individuals’ subjective experiences and perceptions of their EF in real-world contexts, serving as an important complement by offering different, yet equally significant, types of information. Importantly, self-report measures may be better suited to capture the “hot” components of EF, including emotional and motivational aspects that are often underrepresented in traditional, laboratory-based assessments targeting primarily “cold” cognitive processes such as working memory and inhibitory control [25, 26].

Research has consistently found little to no associations between self-reported evaluations of people’s own EF and performance-based measures, underscoring the importance of employing both approaches [27, 28]. Together, these methods may provide a more comprehensive understanding of how EF is related to procrastination by capturing both subjectively perceived and performance-based aspects.

Most facets of EF are relevant to the problem of procrastination. For instance, impulsivity is closely related to procrastination [1], partly through ineffective goal-management [29]. Resisting temptations engages inhibition, the capacity to suppress impulsive, automatic responses [23]. Working memory supports maintaining and updating information about the planned task and goal. Cognitive flexibility, or shifting, enables task initiation and the reallocation of attention. Finally, self-monitoring and metacognitive abilities help evaluate progress and identify when adjustment of strategies are necessary. Together, these EF components help translate intentions into action, and when they fail, procrastination becomes more likely.

However, there is limited empirical evidence on the associations between traditional neurocognitive tests and procrastination [5, 30]. Previous studies were largely performed in academic settings with students, providing indications that self-reported executive functions (EF) are indeed associated with procrastination [5, 30–34]. Rinaldi et al. [30] also found a significant negative association between procrastination and cognitive flexibility, both through self-report measures and in performance-based assessment (Trial Making Test B).

Other psychological constructs that are related to procrastination are certain personality traits, depressive symptoms, and rumination. These are important to consider because they may confound or influence the relationship between procrastination and other variables. We will briefly discuss these in the following.

Procrastination appears to be relatively stable across the lifespan and is consistently associated with personality traits relevant to self-regulation, most notably low conscientiousness and high impulsivity, the latter being a facet of neuroticism [1, 2, 30, 35–39]. Openness to experience, agreeableness, and extraversion have demonstrated weaker and less consistent associations with procrastination [30, 1, 37, 40].

Procrastination is associated with depression [1, 32, 40], possibly via reduced energy levels. Flett et al. [41] found that ruminative brooding, a common depressive symptom, correlated with procrastination and with procrastination-related automatic thoughts, suggesting that procrastinators do not only experience self-regulation failures, but also difficulties with mood regulation. Depression is also linked to poorer EF [27, 30, 32, 42]. When cold EF processes such as attention, working memory, inhibition, and evaluative control are weakened, hot cognitive biases become more influential. Negative mood intensifies these deficits, fostering maladaptive emotion regulation through rumination [42]. Thus, both depression and rumination are important constructs to include when studying EF and procrastination.

The relative roles of hot versus cold EF in procrastination remain unclear. As procrastination theories as the TMT and the Mood Repair Model emphasize impulsivity and impaired cognitive control as central mechanisms underlying procrastination, both hot and cold EF may be related to procrastination. Difficulties with emotion regulation have been suggested as a mediator between fear of failure and procrastination [43] and should be related to hot processes [41, 42]. Conversely, possessing good cold EF should support strategies to overcome the impulsive hot processes, and help regulate behavior towards long-term goals [17].

In the present study, we investigate the associations between procrastination and executive functions in an adult sample with a broad age range. The study incorporates both performance-based and self-reported EF measures, controlled for relevant demographic variables, in addition to depression, rumination, and personality traits. Hence, the study explores whether objective executive deficits or subjective perceptions of executive failures are more strongly associated with procrastination.

Research questions and hypotheses:

1. Are difficulties in executive functioning (both self-reported and performance-based) associated with procrastination in a healthy adult sample.?
2. Which type of EF measure predicts procrastination best when controlling for personality, depression, rumination and demographic factors?

Based on existing empirical evidence, the following hypotheses were formulated:

- H1) Self-reported executive functioning difficulties in daily life will be positively associated with procrastination.
- H2) Performance-based executive function measures will show weak or no associations with procrastination.
- H3) The association between self-reported executive functioning difficulties and procrastination will remain significant when controlling for personality traits (conscientiousness and neuroticism), depressive symptoms, rumination, and demographic variables.

Given the limited and inconsistent empirical evidence regarding the relative importance of specific executive function domains, additional analyses were conducted in an exploratory manner to examine which self-reported executive function subscales are most strongly associated with procrastination.

## Methods

The present study employed a prospective, observational cross-sectional design, using a convenience sample and a single measurement point.

### Participants

A total of 63 women and 45 men (*N* = 108) provided informed consent and participated in the study. All participants completed neuropsychological tests and questionnaires measuring personality traits, procrastination, and executive functions. Demographic characteristics included sex, age, educational level (years of education), and current occupational status (student or employed professional). Of the total sample, 39 participants (35%) were currently enrolled in academic studies. The average age of participants was 36.2 years (range = 22–65), and the mean number of years of education was 17 (*SD* = 2.4; range = 11–26). In the Norwegian educational system, 16 years of education would correspond to a completed bachelor’s degree; 18 to a completed master’s degree. Exclusion criteria included any psychiatric diagnosis, current depressive symptoms, somatic illness requiring ongoing treatment, or a history of brain injury or neurological disease.

### Instruments

Procrastination was measured using the Irrational Procrastination Scale (IPS). The IPS consists of nine items assessing irrational delay [44], and is administered using a five-point Likert scale. Higher scores indicate greater levels of procrastination behavior. Example items are *I put things off so long that my well-being or efficiency unnecessarily suffers*; *I delay tasks beyond what is reasonable*. We used a six-item version, omitting three reverse-scored items that have been shown to be less effective in measuring the procrastination construct [45]. The IPS have demonstrated satisfactory reliability and validity [44–46]. Cronbach’s alpha for six items in this sample was 0.92.

An estimate of verbal cognitive ability was assessed to determine whether the sample fell within the normal range of cognitive functioning and to provide a reference for expected performance on executive function tasks, which often involve verbal mediation. It was measured using the Similarities subtest from the Wechsler Adult Intelligence Scale–Fourth Edition (WAIS-IV; [47]).

#### Performance-based executive function measures

Several aspects of executive functions were measured using performance-based, standardized neuropsychological tests commonly used in clinical research. We selected subtests from the Delis Kaplan Executive Function system [48, 49] to measure inhibition (Color Word Interference Test condition 3; CWIT-3), verbal fluency (FAS), and cognitive flexibility (Trail Making Test, condition 4; TMT-4).

CWIT-3 measures inhibition of automatic verbal responses. In this condition, participants are shown color words printed in incongruent ink colors, and they are asked to name the ink color while inhibiting the more automatic response of reading the written word (i.e., the Stroop effect). The outcome variable was completion time in seconds, where shorter times indicated better inhibitory control.

The verbal fluency test consists of three conditions (letter, category, and category switching), of which we use the letter (phonemic) fluency condition. In brief, participants are asked to say as many words as possible starting with the letters F, A, and S, with a 60 s time limit for each letter. The outcome variable was the total number of correct words produced across the three letter conditions, with repetitions and proper nouns excluded according to standard scoring procedures.

The D-KEFS version of TMT-4 is a paper-and-pen test with five different conditions. In this study, we used condition 4 (also known as TMT-B), which is a number-letter sequencing test, regarded as a sensitive measure of cognitive flexibility and set-shifting. Participants are asked to draw a line, alternating between numbers and letters in serial order (1 – A – 2 – B, etc.). The outcome variable was completion time in seconds, where shorter times indicate better performance.

We also assessed cognitive shifting and problem-solving abilities using the Wisconsin Card Sorting Test (WCST-64:CV2-Research Edition;, [50]). Participants are asked to sort response cards according to changing rules based on color, form or number, without being told about the correct sorting principle. Feedback after each response indicates if the sorting was correct, allowing participants to infer and adapt to new rules as they change throughout the test. In this study, the total number of errors on the WCST was used. Higher scores indicate poorer performance.

Additionally, working memory capacity was measured through a computerized N-back task [51, 52]. In this task, a series of letters appeared sequentially on the screen. In the 0-back condition, participants respond whenever they see the target letter “X”, serving as a baseline measure on attention and reaction time. In the 2-back condition, they respond when the current letter is equal to the one presented two trials earlier, requiring ongoing monitoring and updating information in working memory. Stimuli consisted of letters A-Z and were presented on the pc-screen for a duration of 1000ms, while intervals between stimuli (blank screen) were 2000ms in all n-back tasks. Participants’ responses were registered during the presentation of the stimulus, as well as the first 1000ms of the interstimulus interval. The trials had a fixed order starting with 0-back followed by 2-back. As the independent variable from this test, we used the d-prime value (*d’*) from the 2-back condition, reflecting sensitivity in distinguishing targets from non-targets.

#### Self-report executive function measures

The BRIEF-A (Behavior Rating Inventory of Executive Function - Adult Version) is a self-report questionnaire designed to assess EF in adults [26, 53]. The BRIEF-A consists of 75 items assessing the individual’s behavior over the past month, with response options of “Never,” “Sometimes,” and “Often.” Higher scores indicate greater difficulties. Traditionally, the subscales for Inhibit, Shift, Emotional Control, and Self-Monitoring are combined into the *Behavioral Regulation Index* (BRI), while the subscales for Initiate, Working Memory, Plan/Organize, Task Monitor, and Organization of materials contribute to the *Metacognition Index* (MI). Together, the nine clinical subscales form the *Global Executive Composite* (GEC), which reflects overall executive functioning. However, in the newer versions of the BRIEF used in the US, the BRIEF2 [54, 55], a three-factor solution divides the BRI in two. The subscales Inhibit and Self-monitoring still make up *Behavioral Regulation*, while Shift and Emotional Control comprise the new factor *Emotional Regulation* [26]. Support for the three-factor solution has been demonstrated across different populations and versions of BRIEF [25, 26, 56–59]. We used the official Norwegian translation with respective normative data that are based on a large American sample. Cronbach’s alpha as reported in the manual ranges from 0.73 to 0.90 for the different subscales [53].

#### Covariates

The BFI-10 (Big Five Inventory-10) is a brief 10-item questionnaire designed to assess the five major dimensions of personality: openness to experience, conscientiousness, extraversion, agreeableness, and neuroticism. Each of the five traits is measured by two items, with respondents rating each statement on a 5-point scale from “Strongly disagree” to “Strongly agree.” [60]. Inter-item correlations for the five factors are 0.55, 0.21, 0.56, 0.41, and 0.44, respectively. It is important to note that the items in the BFI-10 were originally selected to represent the breadth of the full scale rather than to achieve high internal consistency between the two items within each factor [60]. As a result, lower reliability scores are to be expected.

BDI-II (Beck Depression Inventory-II) is a self-report questionnaire for measurement of depressive symptoms, with 21 statements that are rated on a 4-point scale [61]. A sum score > 14 is often used as an indication for mild depression [61, 62]. Cronbach’s alpha in this sample was 0.90.

Rumination was measured by the Ruminative Response Scale [63]. It consists of 22 items, response format 1–4 from almost never to almost always. Example items are *Think “I won’t be able to concentrate if I keep feeling this way*”; *Think about all your shortcomings*,* failings*,* faults*,* mistakes*. Cronbach’s alpha in this sample was 0.95.

### Procedure

Data collection occurred between June 2022 and May 2025 at the Department of Psychology, UiT The Arctic University of Norway, Tromsø. The data were collected as part of a larger, ongoing study that functions as a control group for baseline measures in a randomized, double-blind controlled trial investigating the effects of intermittent theta-burst stimulation on depression (ClinicalTrials.gov identifier: NCT05516095; registration date August 25, 2022). This control study was approved by the Regional Committee for Medical and Health Research Ethics, North (Reference: 2022/228765).

Participants were recruited through advertisements posted on university bulletin boards and campus notice boards, as well as through social media and the researchers’ professional networks. Inclusion criteria required participants to be between the ages of 22 and 65, have Norwegian as their native language, and no reported history of depression, other mental disorders, or neurological conditions. Mental and physical health status were assessed via self-report.

All participants were assessed individually in a neuropsychological laboratory. Informed consent was obtained prior to participation. Each participant received a gift card valued at 100 NOK (approximately 10 USD) as compensation. The assessment lasted approximately 1.5 h and included administration of questionnaires and neuropsychological tests. All assessments were conducted by either a licensed clinical neuropsychologist or a trained psychology student under supervision.

### Data Analysis

Neuropsychological tests were scored by the respective published manuals, with age-based normative corrections applied where applicable. An exception was the N-back task, for which an in-house version was used (see [64]). The BRIEF-A was also scored per the manual, with age adjustments applied.

The procrastination variable was assessed for linearity and normality by visual inspection of Q-Q and boxplots and deemed eligible for parametric modelling. Skewness and kurtosis were within acceptable range (0.37 and − 0.65, respectively). Bivariate associations between procrastination and the independent variables were examined using Pearson’s correlation coefficients (H1 and H2).

Preliminary analyses were conducted to ensure no violation of the assumptions of normality, linearity, and homoscedasticity. Residual plots were used to detect outliers, and datapoints ± 3 standard deviations from the mean were a-priori considered outliers. Missing data were considered missing at random, and no imputation of missing data was performed.

To identify predictors of procrastination, hierarchical multiple regression analyses were performed (H3). In the regression analyses of procrastination predictors, we started by including the three BRIEF-A factors along with the five performance-based tests. In the second model, we kept the significant predictors from the first step and added depression, rumination, and the two personality traits known to be associated with procrastination (conscientiousness and neuroticism). Finally, the third model incorporated demographic factors. The regression analysis revealed no issues with multicollinearity, and all VIF values were below 1.7.

Analyses were performed with IBM SPSS Statistics version 29. An alpha-level of *p*<.05 was used as the criterion for statistical significance in all analyses.

## Results

### Descriptives

When describing the sample, we used T-scores/scaled scores for the EF measures; in the analyses, we used the unadjusted raw scores. Descriptive statistics for all variables are shown in Table 1.

**Table 1:** Descriptive Data for all Variables

Measure (range)	Mean	Standard Deviation
QUESTIONNAIRES		
IPS Procrastination (1–5)	2.96	0.65
BDI-II Depression (0–21)	5.45	6.71
Rumination (1–4)	1.55	0.57
BFI-10 Conscientiousness (1–5)	3.74	0.89
BFI-10 Neuroticism (1–5)	2.36	0.90
BFI-10 Extraversion (1–5)	3.65	1.03
BFI-10 Agreeableness (1–5)	4.09	0.78
BFI-10 Openness (1–5)	3.44	0.93
SELF-REPORTED EF		
BRIEF-A Inhibition	50.17	8.38
BRIEF-A Self Monitoring	48.92	9.77
BRIEF-A Shifting	50.65	8.79
BRIEF-A Emotional Control	49.96	10.22
BRIEF-A Initiation	55.04	9.13
BRIEF-A Working Memory	54.48	9.75
BRIEF-A Plan/Organize	53.31	9.25
BRIEF-A Task Monitoring	54.87	8.78
BRIEF-A Organization of materials	50.78	9.08
BRIEF-A Behavior regulation (BR)	49.60	8.06
BRIEF-A Emotional Regulation (ER)	50.30	8.51
BRIEF-A Metacognition index (MI)	54.14	8.80
BRIEF-A General Executive Composite (GEC)	52.50	8.76
PERFORMANCE-BASED EF		
WCST number of errors	49.83	10.57
D-KEFS Color-Word Interference Test condition 3	10.92	1.63
D-KEFS Trail Making Test condition 4	10.33	2.67
Verbal Fluency (FAS)	12.61	3.68
2-back *d’*	3.90	0.54
VERBAL REASONING		
Similarities	13.89	3.37

*IPS *Irrational Procrastination Scale, *BFI-10 *Big Five Inventory–10 items, *BDI-II * Beck Depression Inventory-II, *EF *Executive function, *BRIEF-A *Behavior Rating Inventory of Executive Function - Adult version, *WCST *Wisconsin Card Sorting Test, *D-KEFS *Delis-Kaplan Executive Function System

T-scores are presented for the BRIEF-A and WCST (*M* = 50, *SD* = 10), scaled scores for the D-KEFS tests (*M* = 10, *SD* = 3); raw scores for the rest of the variables.

*N* = 113 for all instruments except IPS (110), TMT-4 and WCST (112) and N-back (98)

### Associations and predictions

As shown in Table 1, the Similarities subtest from the WAIS-IV yielded a mean scaled score of 13.89 (*SD* = 3.37), consistent with high average verbal reasoning ability. Procrastination scores were comparable with what is commonly reported in healthy samples [44–46]. Performance based EF test showed that the participants functioned within the normal to high average range across tests, indicating no clinically significant executive impairments. Consistent with the high educational level in this sample, the BRIEF-A T-scores also indicated average executive functioning across all domains. Slightly elevated scores on the Initiate and Working Memory subscales were observed, yet they were still well within the normal range. The average level of self-reported depression symptoms was low [61].

Correlational data on all variables are presented in Tables 2, 3 and 4. Seven of the nine clinical scales of BRIEF-A were significantly associated with procrastination – the only scales not significantly correlated was Self-Monitoring and Shifting, confirming to a large degree H1. It is noteworthy that the clinical scales on the BRIEF-A most strongly correlated with procrastination were Initiation and, to a slightly lesser extent, Working Memory and Planning/Organization. Both the General Executive Composite and the three factors correlated significantly with procrastination, with Metacognition Index having the strongest and Emotional Regulation the weakest coefficients.

**Table 2:** Correlations between procrastination and BRIEF-A scales and indices

Variable	1	2	3	4	5	6	7	8	9	10	11	12	13
1. Procrastination													
2. Inhibition	0.39***												
3. Self-Monitoring	0.16	0.56***											
4. Shifting	0.32	0.41***	0.31***										
5. Emotional Control	0.29**	0.47***	0.38***	0.56***									
6. Initiating	0.75***	0.56***	0.23*	0.46***	0.41***								
7. Working memory	0.57***	0.70***	0.50***	0.54***	0.46***	0.64***							
8. Plan/Organize	0.62***	0.59***	0.42***	0.45***	0.40***	0.70***	0.70***						
9. Task Monitoring	0.43***	0.48***	0.33***	0.44***	0.41***	0.51***	0.54***	0.60***					
10. Organization	0.46***	0.39***	0.16	0.34***	0.40***	0.58***	0.41***	0.51***	0.52***				
11. BR	0.32***	0.90***	0.86***	0.41***	0.49***	0.46***	0.69***	0.58***	0.47***	0.32***			
12. ER	0.34***	0.50***	0.40***	0.79***	0.95***	0.47***	0.55***	0.46***	0.47***	0.42***	0.51***		
13. MI	0.67***	0.67***	0.38***	0.55***	0.51***	0.85***	0.79***	0.87***	0.74***	0.76***	0.61***	0.59***	
14. GEC	0.60***	0.77***	0.55***	0.67***	0.73***	0.77***	0.82***	0.82***	0.70***	0.68***	0.76***	0.79***	0.94***

Significance levels: * *p* < .05, ** *p* < .01, *** *p* < .001. *BR *Behavioral Regulation, *ER *Emotional Regulation, *MI *Metacognition Index, *GEC *General Executive Composite

**Table 3:** Correlations between procrastination and performance-based EF tests

Variable	1	2	3	4	5
1. Procrastination					
2. WCST total errors	− 0.05				
3. CWIT-3	0.04	0.14			
4. Verbal Fluency	0.04	− 0.22*	0.06		
5. TMT-4	− 0.06	0.17	0.09	− 0.14	
6. 2-back *d’*	− 0.09	− 0.05	− 0.08	0.09	− 0.22*

Significance levels: * *p* < .05, ** *p* < .01, *** *p* < .001

*WCST*  Wisconsin Card Sorting Test, *CWIT-3* Color Word Interference Test condition 3, *TMT-4* Trail Making Test condition 4

**Table 4:** Correlations between procrastination and covariates

Variable	1	2	3	4	5	6	7	8	9	10	11	12
1. Procrastination												
2. Age	− 0.18											
3. Sex	− 0.11	− 0.18										
4. Education	− 0.04	0.29**	− 0.12									
5. Student status	− 0.08	0.59***	− 0.08	0.42***								
6. Depression	0.40***	− 0.11	− 0.07	− 0.15	− 0.19*							
7. Rumination	0.39***	− 0.19*	− 0.32***	− 0.08	− 0.20*	0.64***						
8. Conscientiousness	− 0.60***	0.04	0.02	0.08	0.08	− 0.34***	− 0.23**					
9. Neuroticism	0.25**	− 0.19*	− 0.34***	0.07	− 0.08	0.39***	0.54***	− 0.20*				
10. Extraversion	− 0.10	0.08	0.17	− 0.04	− 0.08	− 0.10	− 0.26**	0.05	− 0.38***			
11. Agreeableness	0.16	0.15	− 0.22*	0.13	0.14	− 0.21*	− 0.11	− 0.11	− 0.10	0.08		
12. Openness	0.04	0.02	− 0.01	0.04	0.02	− 0.10	− 0.09	0.06	− 0.04	0.04	− 0.12	
13. Similarities	0.08	0.19*	− 0.06	0.39***	0.15	− 0.09	− 0.04	− 0.03	− 0.04	− 0.06	0.24*	− 0.05

Significance levels: * *p* < .05, ** *p* < .01, *** *p* < .001

No significant associations were found between procrastination and performance-based EF tests, as expected (H2). Regarding personality traits, both neuroticism and conscientiousness, as measured by the BFI-10, were significantly correlated with procrastination. The remaining personality traits did not demonstrate significant associations. 

The main regression analysis is shown in Table 5. First, we included all measures of EF, i.e., the three BRIEF-A factors, and the five performance-based tests. Only the Metacognition Index was a significant predictor of procrastination. In the next step, we therefore removed all other variables and included depression, rumination, conscientiousness, and neuroticism. BRIEF-A MI was still significant together with conscientiousness. In the final step, we kept these two, while also including all demographic variables (age, sex, length of education and status as a student) as control variables.

**Table 5:** Predictors of procrastination

Variable	B	SE B	BETA	t	*p*	*R* ^2^
*Model 1*						0.47
BRIEF-A Behavioral regulation	− 0.03	0.02	− 0.12	-1.18	0.243	
BRIEF-A Emotional regulation	− 0.00	0.03	− 0.01	-0.13	0.896	
BRIEF-A Metacognition index	0.06	0.01	0.74	6.90	< 0.001	
WCST total errors	− 0.00	0.01	− 0.01	-0.15	0.887	
CWIT-3	− 0.01	0.01	− 0.10	-1.08	0.285	
Verbal Fluency	0.00	0.01	0.00	0.05	0.960	
TMT-4	− 0.00	0.00	− 0.05	− 0.56	0.575	
2-back *d’*	0.03	0.13	0.02	0.23	0.822	
*Model 2*						0.53
BRIEF-A Metacognition index	0.04	0.01	0.45	4.94	< 0.001	
Depression	− 0.00	0.01	− 0.01	-0.07	0.948	
Rumination	0.01	0.01	0.16	1.64	0.105	
Conscientiousness	− 0.31	0.08	− 0.30	-3.62	< 0.001	
Neuroticism	0.03	0.08	0.03	0.42	0.679	
*Model 3*						0.55
BRIEF-A Metacognition index	0.04	0.01	0.48	5.78	< 0.001	
Conscientiousness	− 0.32	0.08	− 0.31	-3.79	< 0.001	
Age	− 0.01	0.01	− 0.19	-2.27	0.025	
Sex (female = 1, male = 2)	− 0.22	0.13	− 0.12	-1.74	0.085	
Education in years	0.02	0.03	0.05	-0.71	0.482	
Student (student = 1, other = 2)	0.16	0.16	0.85	-0.98	0.329	

*WCST *Wisconsin Card Sorting Test, *CWIT *Color Word Interference Test condition 3, *TMT-4 *Trail Making Test condition 4

The final regression model explained 55% of the variance in the procrastination measure (adjusted *R*^*2*^=0.52). The final significant predictors were the BRIEF-A Metacognition Index, conscientiousness, and age. This partly confirms H3.

To further explore research question 2 - which subscales in the metacognition index contribute most to predicting procrastination - we performed an additional regression analysis (Table 6). As Initiate correlated most strongly with procrastination, we first included the other four subscales. Both Working memory, Plan/Organize and Organization significantly predicted procrastination, while Task Monitoring did not. Next, we included Initiate, which rendered all other subscales non-significant. Finally, we included Conscientiousness and age as covariates. Initiate and Conscientiousness predicted procrastination, and the final model explained 61% of the variance (adjusted *R*^*2*^=0.58).

**Table 6:** Predictors of procrastination, metacognitive subscales

Variable	B	SE B	BETA	t	*p*	*R* ^2^
*Model 1*						0.47
Working memory	0.08	0.03	0.25	2.40	0.018	
Plan/Organize	0.10	0.03	0.35	3.06	0.003	
Task monitoring	− 0.01	0.04	− 0.02	-0.16	0.872	
Organization	0.05	0.03	0.18	2.01	0.048	
*Model 2*						0.58
Working memory	0.03	0.03	0.10	1.02	0.308	
Plan/Organize	0.03	0.03	0.12	1.14	0.255	
Task monitoring	− 0.01	0.04	− 0.02	-0.19	0.854	
Organization	0.01	0.02	0.02	0.25	0.801	
Initiate	0.19	0.03	0.60	5.95	< 0.001	
*Model 3*						0.61
Working memory	0.04	0.03	0.12	1.25	0.215	
Plan/Organize	0.02	0.03	0.06	0.58	0.563	
Task monitoring	− 0.01	0.04	− 0.03	-0.33	0.739	
Organization	0.00	0.02	0.01	0.06	0.953	
Initiate	0.16	0.03	0.50	4.79	< 0.001	
Conscientiousness	− 0.23	0.08	− 0.23	-2.79	< 0.001	
Age	− 0.00	0.01	− 0.05	-0.76	0.451	

## Discussion

The present study adds to the literature in two ways: First, it identifies Initiation as the strongest and most specific metacognitive predictor of procrastination in adults. Second, it is the first to apply the three factor BRIEF-A structure in procrastination research.

We examined the relationship between executive functions (EF) and procrastination using both self-report and performance-based measures in a non-clinical adult sample. We hypothesized that self-reported executive functioning difficulties in daily life would be positively associated with procrastination (H1), but that performance-based measures would show weak or no associations with procrastination (H2). Our main findings confirm H1 and show that greater self-reported difficulties with EF in daily life, particularly metacognitive problems with initiating tasks, were associated with increased procrastination. Scores on the performance-based tests were not, confirming H2. In line with previous research, conscientiousness and neuroticism correlated with procrastination, as did symptoms of depression and rumination. Of the demographic variables, only age was a significant predictor, with older participants reporting less procrastination.

H3 stated that the association between self-reported executive functioning difficulties and procrastination would remain significant when controlling for personality traits (conscientiousness and neuroticism), depressive symptoms, rumination, and demographic variables.

The results showed that the BRIEF Metacognition Index significantly predicted procrastination after including the other variables, but not the Behavioral and Emotional Regulation indices or the performance-based measures, only partly confirming H3. Furthermore, conscientiousness was a significant predictor in the regression model, but not neuroticism, which has been linked to procrastination in prior research [1, 30, 37, 40]. This discrepancy may be due to the inclusion of depression and rumination as covariates, which could account for the emotional instability typically associated with neuroticism. However, depression or rumination did not predict procrastination either, despite significant bivariate associations found in correlational analyses. The predictors in the final model together explained 55% of the variance in the procrastination variable suggesting that metacognitive executive difficulties and low conscientiousness are robust, independent correlates of procrastination in adults.

Our second, exploratory, research question was to further clarify which specific aspects of metacognitive functioning are most relevant to procrastination through examining the individual BRIEF-A subscales. All subscales within the Metacognition index–Initiate, Plan/organize, Working memory, Task monitor and Organization of materials–demonstrated moderate correlations with procrastination, in line with previous research [32, 34]. In contrast, Inhibit and Emotional Control showed only small correlations, while Shift and Self-monitor were not significantly related to procrastination. In a regression model, the subscale Initiate outperformed all other subscales.

Hence, a central contribution of this study is the demonstration that initiation difficulties, as measured with the BRIEF-A Initiation subscale, showed a unique and strong association with procrastination, overshadowing all other EF components once included in the model. This aligns with the contemporary conceptualization of procrastination as failure to act on intentions in a timely manner, and underscores how postponing the start of an intended action – the intention-action gap - is a core feature of procrastination [6, 65]. This failure is particularly evident when individuals face aversive [3] or abstract tasks [66].

Initiation difficulties fit well with process models of procrastination, such as Temporal Motivation Theory [7]. TMT emphasizes that procrastination occurs when the perceived utility of a task is low, which is often the case with abstract or aversive tasks. The strong relationship between procrastination and the Initiate subscale may therefore reflect difficulties in overcoming the low immediate motivation connected to such tasks. This may be explained by a cognitive bias that makes postponing a task seem significantly less effortful while offering nearly the same level of reward as completing it immediately [67]. Both constructs – procrastination and the ability to initiate an action – are closely tied to self-regulation and motivation, as they involve the capacity to manage behavior in pursuit of a goal. However, it is important to note that the ability to initiate an action represents only one of several processes associated with procrastination, which is a broader and more complex phenomenon. Clarifying the specific role and importance of this process within the broader framework of procrastination is therefore of significant interest.

A second novel contribution of the study is the use of the new three-factor BRIEF-A model in relation to procrastination. Prior research has relied largely on two-factor or global indices. The Emotional Regulation factor, which reflects hot aspects of EF, did indeed correlate positively with procrastination, suggesting that difficulties in managing emotions are associated with more procrastination. However, when ER was included in a regression model alongside the other BRIEF factors, meta-cognitive abilities emerged as the stronger predictor, rendering ER non-significant. The low correlation between ER and procrastination may indicate that emotional regulation plays a less direct role, but difficulties in task initiation (reflected in Initiate) could also serve to avoid negative emotions, in line with the Mood Repair Theory [3]. This distinction supports the view that procrastination is a failure of goal management and not emotional dysregulation alone.

Overall, the findings highlight the importance of metacognitive abilities, and especially the ability to initiate a task, as a key factor in understanding procrastination, consistent with prior studies [30, 32, 34]. Metacognition, which involves planning, organizing, and monitoring goal-directed behavior, is essential for developing effective plans and turning them into action [18, 23]. Specifically, the results suggest that individuals with greater metacognitive difficulties may struggle to manage competing demands, prioritize long-term goals, and resist immediate temptations, leading to procrastination.

The lack of a relationship with performance-based executive function tasks suggests a potential disconnect between cognitive ability measured in tests and how people actually function in real-life situations. We are aware of only two previous studies that used a broad clinical battery of performance-based tests to examine multiple aspects of EF in relation to procrastination [30, 36]. In the current study, none of the performance-based EF tests showed a significant correlation with procrastination. As a result, we were unable to replicate the findings reported by Rinaldi et al. [30], who suggested that the Trail Making Test Part B (TMT-B) could serve as a quick screening tool for identifying individuals prone to procrastination. This discrepancy may be due to differences in the study samples, as Rinaldi et al. focused on college students only. Additionally, Rinaldi used a different version of the TMT test than the one used in the present study (TMT-4 from the D-KEFS test battery). They also reported that their sample performed significantly below expectations on the TMT-B, whereas our participants performed at or above average levels. Gustavson et al. [36] found a significant association between procrastination and one of three inhibition tests (the stop signal task), but not on the color-word task. Furthermore, both Rinaldi et al. [30] and Gustavson et al. [36] primarily found only small or close-to-zero correlations between the performance-based measures and procrastination.

One plausible interpretation of the discrepancy between self-report and objective tests is that the BRIEF-A has better ecological validity, as it captures EF as it is experienced in everyday life, including both cold and hot aspects of EF [25, 26]. The BRIEF-A reflects individuals’ ability to plan, organize, and initiate behavior in complex, real-world settings. This ecological validity could help explain the stronger correlations observed between BRIEF-A scores and procrastination, in contrast to the lack of association found with traditional, laboratory-based executive function tests. especially in high-functioning adult samples.

The present study has several notable strengths. First, the procrastination measure employed captures a broad conceptualization of procrastinatory behavior, not limited to academic procrastination, increasing its ecological validity. Second, the study utilized an extensive and well-validated battery of cognitive tests, covering a wide range of EF domains with instruments commonly used in clinical neuropsychology. The performance-based tests are supported by normative data, providing a robust comparison group and allowing for reliable interpretation of individual differences. Also, this study used *both* performance-based and self-reported EF as predictors of procrastination to clarify their differential roles in procrastination. This highlights an important issue that has received little attention in procrastination research, namely, the discrepancy between performance-based assessments and self-report measures of executive function.

Importantly, relatively few studies have examined the relationship between procrastination and clinically validated neuropsychological EF tests. In this regard, the present study makes a unique contribution by offering a comprehensive assessment of EF beyond self-report instruments alone. Furthermore, unlike many previous studies in the field, which often rely exclusively on young student populations, the current sample includes a broader age range and a more diverse group of adults. This enhances the generalizability of the findings and contributes to a more representative understanding of procrastination in the general population. Finally, to the best of our knowledge, this is the first study to use the new, three-factor model of BRIEF-A to investigate the associations between self-report EF and procrastination, contributing to our understanding of procrastination related to the cold, as opposed to hot, EF.

Some limitations are worth mentioning. First, the design does not allow us to draw any causal conclusions regarding the associations between variables. It is also, of course, possible that the selection of different neuropsychological tests or other measurements of personality and procrastination might have yielded different results. Experimental neuropsychological tasks, particularly those designed to probe specific cognitive mechanisms, are likely to be more sensitive in detecting subtle deficits and elucidating the cognitive underpinnings of procrastination. In contrast, standard clinical EF tests may lack the sensitivity needed to capture variation within non-clinical or high-functioning samples. Self-report questionnaires, while valuable in assessing everyday executive challenges, are prone to various biases, including mood-related distortions and personality influences, which complicate interpretation. It is also possible that a self-selection bias among participants could influence the results. Procrastinators may be less likely to volunteer for research studies than others, because of commitment avoidance or decision postponement (e.g., [1]). Conversely, the opposite could also be true: participating in such a study might serve as an appealing form of procrastination, offering an alternative to more pressing tasks like studying, thereby making procrastinators more likely to participate. However, mean scores on procrastination in our sample did not deviate significantly from previous studies [44–46]. Since the data were collected as part of a broader study without procrastination as its primary focus, substantial self-selection effects specifically related to procrastination are considered unlikely.

Our results should also be interpreted in light of the characteristics of the present sample, consisting primarily of individuals with higher education and without diagnosed conditions typically associated with EF difficulties. Participants performed within the normal to high-average range on executive function tests, indicating no clinical impairments. BRIEF-A scores were also average across domains, which is consistent with the group’s high education level. However, slightly higher scores than the normative mean were noted for Initiate and Working Memory. These findings suggest that even cognitively capable individuals may perceive at least some difficulties in starting and maintaining goal-directed activities. These subtle metacognitive difficulties, although within the normative age range, may help explain procrastination in high-functioning adults. Executive functions develop relatively late in life, and the relationship between executive functioning and procrastination may therefore change with age, maturation, and education. It is possible that emotional and motivational aspects of executive functioning are more central to procrastination among younger individuals, such as students, who often operate in less externally structured environments, characterized by greater autonomy and flexible schedules, and they also report higher levels of psychological distress. Metacognitive aspects may become more relevant for older adults in professional work-related contexts, characterized by more external structure, fixed deadlines, and more consistent external regulation. Accordingly, the associations between emotional/hot and cognitive/cold aspects of executive functions and procrastination may be both age and context-dependent, reflecting differences in developmental stage, environmental demands, and mental health burden (e.g., [68]).

Our results have some applied implications. Given the strong association between metacognitive difficulties and procrastination, potential interventions for reducing procrastination should focus on enhancing metacognitive skills such as planning, organization, and self-monitoring to help develop proactive self-regulation strategies [19]. Specifically, interventions that facilitate the initiation of aversive tasks, for instance through reducing fear of failure and increasing self-efficacy for the task [69], or strengthening implementation intentions and minimizing onset-delay [65], may prove particularly useful. Additionally, interventions targeting conscientiousness, such as habit formation and goal-setting exercises, could be effective in reducing procrastination [70].

Further research could include longitudinal studies to examine how changes in different EF over time influence procrastination. Additionally, the role of hot and cold EF in procrastination should be further explored, including using both self-report and behavioral measures of procrastination. For example, studies could investigate how emotional regulation strategies interact with meta-cognitive processes to influence actual procrastination. Testing the associations between procrastination and hot and cold aspects of EF in other populations, including clinical samples, will help shed more light on how different contexts influence these relationships.

## Conclusions

We investigated the relationship between executive functions (EF), as measured by self-report and performance-based tests, and procrastination in a diverse adult sample and across contexts. The findings revealed that self-reported metacognitive difficulties, as measured by the BRIEF-A Metacognition Index, and particularly the Initiate subscale, showed the strongest association with procrastination among the variables examined, after controlling for depression, rumination, and demographic factors. Additionally, low conscientiousness and higher age emerged as significant predictors. Neither the Behavior Regulation and Emotional Regulation indices from BRIEF-A, nor the performance-based EF measures, showed significant associations with procrastination.

## Data Availability

The data that support the findings of this study are available from the University Hospital of North Norway, but restrictions apply to the availability of these data, which were used under license for the current study, and so are not publicly available. Data are however available from the second author upon reasonable request and with permission of the University Hospital of North Norway.

## Abbreviations

BDI-II: Beck Depression Inventory-II
BFI-10: Big Five Inventory-10
BRIEF-A: Behavior Rating Inventory of Executive Function - Adult Version
BR: Behavioral Regulation
CWIT-3: Color-Word Interference Test, condition 3
D-KEFS: Delis-Kaplan Executive Function System
EF: Executive Functions
ER: Emotional Regulation
GEC: Global Executive Composite
IPS: Irrational Procrastination Scale
MI: Metacognition Index
TMT: Temporal Motivation Theory
TMT-4/B: Trail Making Test, condition 4/B
WAIS-IV: Wechsler Adult Intelligence Scale–Fourth Edition
WCST: Wisconsin Card Sorting Test
